# Integrated approach to water resource management in Mashhad Plain, Iran: actor analysis, cognitive mapping, and roadmap development

**DOI:** 10.1038/s41598-023-50697-x

**Published:** 2024-01-02

**Authors:** Mahdi Kolahi, Kamran Davary, Hamid Omranian Khorasani

**Affiliations:** 1 Faculty of Natural Resources and Environment, Water and Environment Research Institute, Ferdowsi University of Mashhad, PO BOX 9177948974, Mashhad, Iran; 2 Faculty of Agriculture, Water and Environment Research Institute, Ferdowsi University of Mashhad, Mashhad, Iran; 3 Innovative Ecosystem Company, Mashhad, Iran

**Keywords:** Environmental sciences, Environmental social sciences

## Abstract

Iran's Mashhad Plain faces a severe water crisis due to the excessive exploitation of groundwater, leading to the depletion of its aquifer. While water demand management is recognized as a superior solution compared to supply projects, its implementation presents notable challenges. This study addresses the urgent necessity to curtail water demand in the Mashhad Plain by alleviating conflicts among various stakeholders, including water resource managers and consumers. Initially, the research identifies key water resource actors who collaborate in devising a comprehensive roadmap and conceptual model for efficient water resource management. An analysis uncovers significant conflicts among actors, representing approximately 6% of identified issues, with minor conflicts in 30% of cases. Encouragingly, stakeholders demonstrate potential for consensus on the remaining conflict items, with specific actors emerging as pivotal in conflict resolution. Efficient water resource management in regions facing scarcity, diverse interests, and sustainability challenges demands a multifaceted strategy. Future endeavors involve developing a dynamic system model to simulate policy impacts and strengthen conflict resolution efforts. This research introduces a roadmap-driven approach aimed at resolving conflicts and implementing water demand management in the Mashhad Plain. It emphasizes the critical need to address water scarcity challenges while effectively mitigating conflicts among water resource stakeholders.

## Introduction

The global population is steadily rising, resulting in an increased demand for water resources^1^. This surge in demand has raised significant concerns, as some regions benefit from abundant water resources while others face acute water scarcity and pollution. The underlying causes of this water crisis can be attributed to mismanagement, governance issues, climate fluctuations, and population growth^2–5^. In recent decades, water authorities have predominantly concentrated on enhancing water supply by diverting resources from other watersheds, rather than focusing on the fundamental aspect of managing water demand^6^. In Iran, the government oversees various aspects of water management, including both supply and demand management. Consequently, policymakers in the water sector primarily assume responsibility for both aspects.

While prioritizing water supply is justifiable in regions endowed with diverse water resources, it falls short in areas grappling with water deficits. In water-scarce regions, the options are significantly limited. The intricate nature of water demand in arid regions has led to escalated infrastructure costs and heightened environmental risks^7,8^. In areas where water transfer is impractical, two main strategies have surfaced to tackle the challenge of water scarcity: water demand management and water conservation. While these terms are often used interchangeably, it is crucial to distinguish between them. Water demand management involves a spectrum of plans and actions aimed at improving water use efficiency, reducing consumption, and ultimately exerting control over and decreasing water demand. Conversely, water conservation focuses on strategies and actions intended to prevent the over-exploitation of water resources^9^.

The management of social system, such as water resources, has assumed unprecedented complexity in recent times. These systems are characterized by their dynamic nature, multi-objective nature, multidimensional aspects, and the participation of a multitude of stakeholders^10^. In this intricate landscape, water managers formulate strategies while water users are subject to these strategies, forming a web of interactions rife with conflicts. This complexity in water resources management arises from several interrelated factors:

- **Multiplicity of Actors in Water Resources Management:** Various stakeholders pursue divergent strategies and policies in their quest to achieve distinct objectives. This diversity extends to their perceptions of available resources and their goals, thereby complicating the process of consensus-building^11^.
- **Multidimensionality of Water Resources Management:** The intricate behavior of social systems, influenced by personal, organizational, and group dynamics, further compounds the challenge of aligning objectives and strategies^12^.
- **Dynamics and Relative Sustainability in Water Resources Management:** The fluidity of goals and regulations governing decision-making necessitates constant adaptation by stakeholders, who draw insights from prior experiences to ensure relative sustainability^13^.
- **Lack of Consumer Protection:** The absence of safeguards for consumers poses a substantial obstacle to effective water management, leading to numerous conflicts^14^. These conflicts often arise from differing viewpoints on water ownership, resource management, food security, and water security^15^. The resolution of these challenges mandates a reevaluation of approaches by both managers and consumers to attain water resource sustainability^16^.
- **Economic and social impacts**: Over-extraction of water from aquifers can have serious effects on the region's economy and society. Reduced water resources can lead to a decline in agricultural production, increased costs, and decreased economic resilience^17^.

In the realm of water resource management, establishing effective communication between managers and water users is imperative^18^. This communication serves as a vehicle to heighten awareness of water resource problems and the potential ramifications of diverse policies^19^. Nevertheless, forging such communication pathways proves to be a formidable task, primarily due to the inherent divergence in perspectives and objectives between these two groups^20^.

Consumers hold a central position in shaping water management programs and are significantly impacted by the strategies imposed upon them. Therefore, accounting for consumer behavior becomes pivotal for achieving success, given that individual actions exert direct and indirect influence on resource consumption^21^. Indeed, the management of natural resources mirrors human resource management, and any lapse at the local decision-making level can result in the failure of broader solutions. In other words, human environmental decisions can lead to natural environmental changes. For instance, increasing water prices as a demand management strategy may inadvertently lead to a surge in illegal water harvesting and subsequently, greater water consumption. Thus, conflicts often emerge from how consumers engage with and are empowered by management programs^22^.

Pricing policies for water can have far-reaching effects on water resource demand. In fact, water pricing directly impacts:

1. Water Consumption Patterns: When water prices are high, demand for water in consumptive activities such as irrigation may decrease. Consumers may attempt to reduce their water consumption or adopt water-saving practices. Moreover, high water prices can serve as an incentive for better utilization of water-saving technologies and equipment.
2. Water Resource Distribution: Water pricing can directly influence the distribution of water resources within communities. If water prices for agricultural purposes are high, farmers might be encouraged to adopt water-efficient agricultural practices or opt for crops that consume less water. Additionally, water pricing can affect the allocation of water resources between regions. For instance, high water prices may incentivize the transfer of water resources from areas with abundance to those facing scarcity.
3. Attitude Toward Water Resources: Water pricing can shape public attitudes regarding the value of water resources. When water prices are excessively low, individuals may perceive water resources as infinite and easily accessible, paying less attention to sustainable consumption and resource preservation. However, appropriately determined water prices might instigate a greater value for conservation and efficient water use.

Moreover, the actions of water managers, tasked with designing and implementing policies, wield substantial influence over consumer behavior^23^. Their personal, organizational, and group beliefs and theories play a pivotal role in shaping the consumption landscape. When these stakeholders prioritize short-term interests over the long-term sustainability of natural resources, the consequences can be resource depletion^24^. Transparent communication and an understanding of the assumptions underlying decision-makers' choices are essential for mitigating conflicts^25^. However, transparency can also serve as the source of conflict if not managed effectively, potentially jeopardizing the sustainability of water resources.

To navigate and resolve these conflicts, the initial step is their identification^26–29^. By comparing the cognitive maps of managers and consumers, these conflicts can be unearthed^30^. Once identified, interactive learning environments can be employed to gain insights into the unforeseen consequences of decision-making. This approach fosters increased interaction between water consumers and managers, ultimately enhancing their understanding of water resource dynamics^31^.

The behavior of social systems further complicates water resources management^32^. Personal, organizational, and group dynamics add layers of complexity to decision-making processes, creating additional hurdles in the quest for alignment of objectives and strategies. Moreover, as goals and regulations governing water resources management are in a constant state of flux, stakeholders must adapt and draw from their experiences to ensure the relative sustainability of resources^33^.

Key actors play a crucial role in any project, often possessing the potential to exert the most significant impact on the project's outcomes or being most affected by the project's results^34^. Moreover, key actors can include those who resist project-induced changes. Managing and maintaining effective communication with key actors throughout the project's lifespan is essential^35,36^. Identifying these key actors is invaluable for obtaining valuable feedback and determining the appropriate level of engagement and communication with them^37^.

Predicting the effects of managerial policies on consumers and resolving their conflicts using cognitive maps enhances actors' understanding of how their behavior impacts water resources. This improved understanding leads to more responsible behavior in conserving water resources. Analyzing and extracting the perspectives of both managers and consumers regarding their current situation aids in comprehending the contradictions and potential resolutions. Various tools, such as the roadmap as a conceptual model for aligning individual perceptions and cognitive maps as conflict identifiers, were employed to predict, prevent, and address conflicts. These tools facilitate the identification of underlying issues and the discovery of mutually beneficial solutions.

Cognitive mapping is a valuable technique for revealing how individuals understand complex systems or subjects^21^. Cognitive maps serve as valuable tools in uncovering shared interests and points of contention, while interactive learning environments facilitate improved comprehension and collaboration among water consumers and managers. It has found applications in diverse decision-making areas, including strategic management^38^ and natural resources management^39^. Cognitive maps offer insights into individual cognitive models and facilitate group strategy development, quantitative information modeling, and structural analysis.

In this study, we employ the cognitive mapping technique to understand the disparities in perceptions between managers and water users and to mitigate conflicts among them. Overall, this research seeks to address the multifaceted challenges surrounding water resources management by exploring conflicts, leveraging local expertise, and formulating strategies for sustainable water demand management.

The principal aim of this study is to discern conflicts among stakeholders, leading to the creation of a framework for water demand management that gains substantial acceptance among stakeholders and can be implemented effectively. To realize this overarching goal, the study aims to achieve the following objectives:

- Identification of water consumers and managers operating within a specific geographical plain.
- Gathering local knowledge from both consumers and experts entrenched in the area.
- Extraction of demand management strategies via collaborative brainstorming sessions involving relevant stakeholders.
- Identification and analysis of existing conflicts and agreements between water consumers and managers.
- Formulation and construction of a conceptual model tailored for demand management within the designated plain.

## Material and methods

### Study area

**Figure 1:**
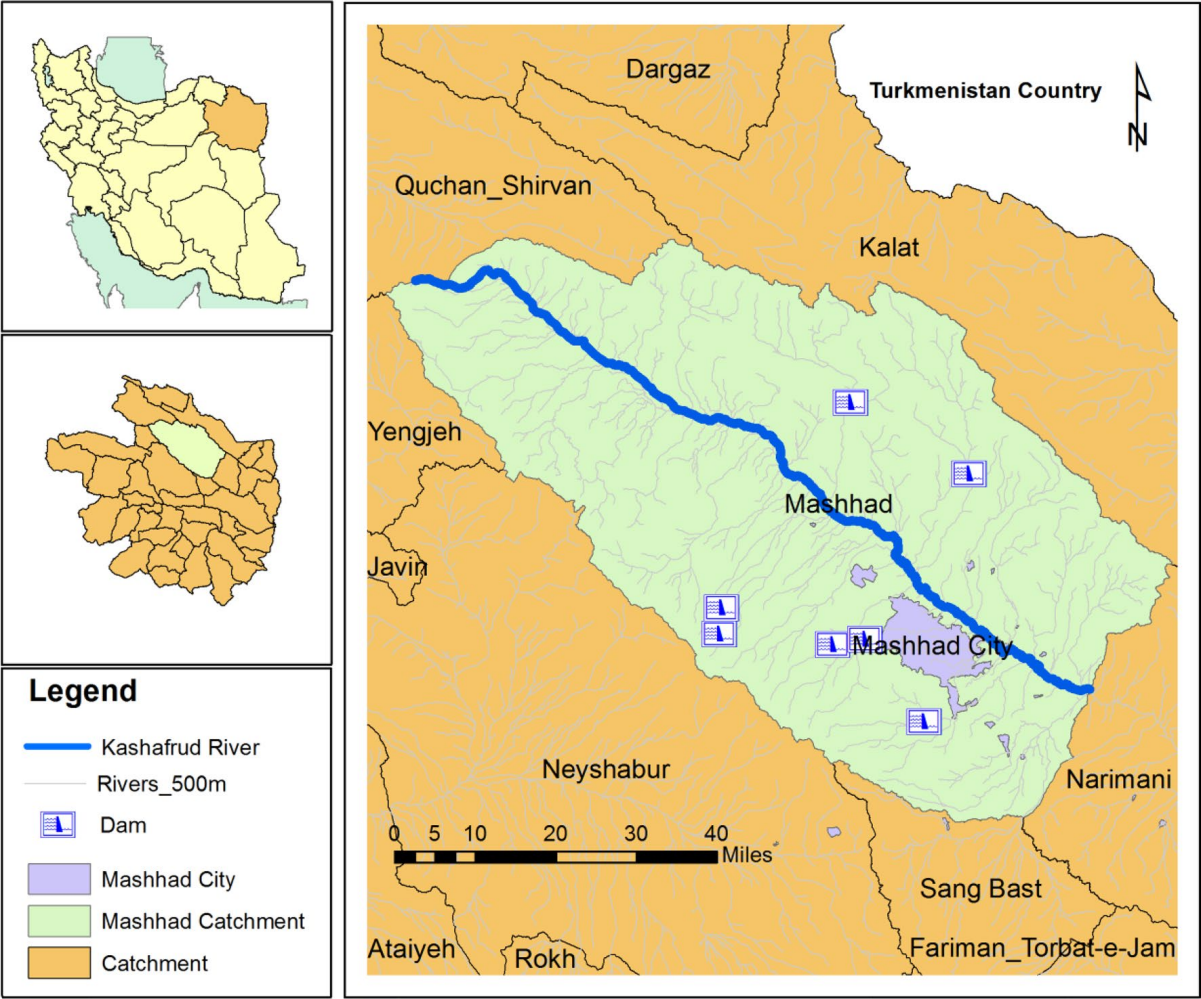
Map of the study area of Mashhad and the locations of cities and villages (ArcGIS10.2, https://www.esri.com/about/newsroom/arcwatch/the-best-of-arcgis-10-2/).

The research focuses on the Mashhad Plain, part of the Kashafroud basin in northeastern Iran's Khorasan Razavi province. Spanning the heart of the plain, the Kashafroud River acts as its primary drainage system (Fig. 1). Over a 30-year statistical period, this region records an average annual rainfall of 261 mm, countered by evaporation and transpiration at 1776 mm. With renewable water volume at 443 million cubic meters, the 30-year underground water reservoir shows an annual deficit of − 82 million cubic meters.

The Mashhad Plain, situated in northeastern Iran, confronts significant water challenges. With agriculture consuming approximately 65% of the water, the city of Mashhad, housing over 3 million residents, emerges as the primary consumer for drinking and services. Moreover, burgeoning industries around Mashhad further intensify water extraction, sparking conflicts among different sectors. Irresponsible water usage diminishes both the quality and quantity of water resources, primarily reliant on groundwater, now dwindling due to imbalanced consumption. Surface water scarcity, coupled with a lack of consensus on water management, amplifies the inefficiency of water management strategies. Hence, a concerted effort aims to align stakeholders’ perspectives and develop a collective roadmap for water resource management.

Incorporating Mashhad and several other cities such as Chenaran, Torghabeh, Shandiz, and Razaviyeh, the plain witnesses an annual precipitation of approximately 2219 million cubic meters. Simultaneously, evaporation and transpiration together account for 1776 million cubic meters. The region's renewable water resources are estimated at 342 million cubic meters per year, with a return flow reaching 443 million cubic meters. At present, the combined consumption of surface and groundwater stands at 927 million cubic meters per year, catering to an approximate population of four million inhabitants^40^. Over the years, water demand from the service and industrial sectors has escalated substantially, surging from 8% in 1971 to 40% in 2015^41^, thereby resulting in a notable annual water balance deficit of more than 70 cm within the aquifer^40^.

The water scarcity issue in the Mashhad Plain is complex and deep-seated, evident in the inadequacy of water transfer solutions. Past efforts, particularly the Doosti Dam water transfer project, intended to alleviate the water scarcity issue, fell significantly short, conveying only around 15 million cubic meters annually due to diplomatic issues between Iran and Afghanistan. Consequently, this sole project failed to resolve the water scarcity problem in the Mashhad Plain^40^.

The region’s water demand-to-supply ratio at approximately 1.3 highlights a pressing challenge stemming from rapid population growth, industrial expansion, and agricultural development. Climate change exacerbates this scenario, potentially decreasing water resource yields while augmenting demand, further diminishing available surface and groundwater reserves.

The depletion of groundwater reserves across Iran is a critical issue impacting aquifers and freshwater supply. A sharp decline in groundwater recharge by approximately 3.8 mm per year between 2002 and 2017 poses a grave threat to providing freshwater to Iran’s population of over 80 million^42^. Groundwater recharge averaging around 40 mm per year surpasses the reported annual surface runoff of approximately 32 mm per year in Iran, emphasizing the vital role of surface waters in recharge. This decline in groundwater recharge within the Mashhad Plain may exacerbate an already critical situation, necessitating urgent water management actions to mitigate environmental and socio-economic repercussions^42^.

### Research process

**Figure 2:**
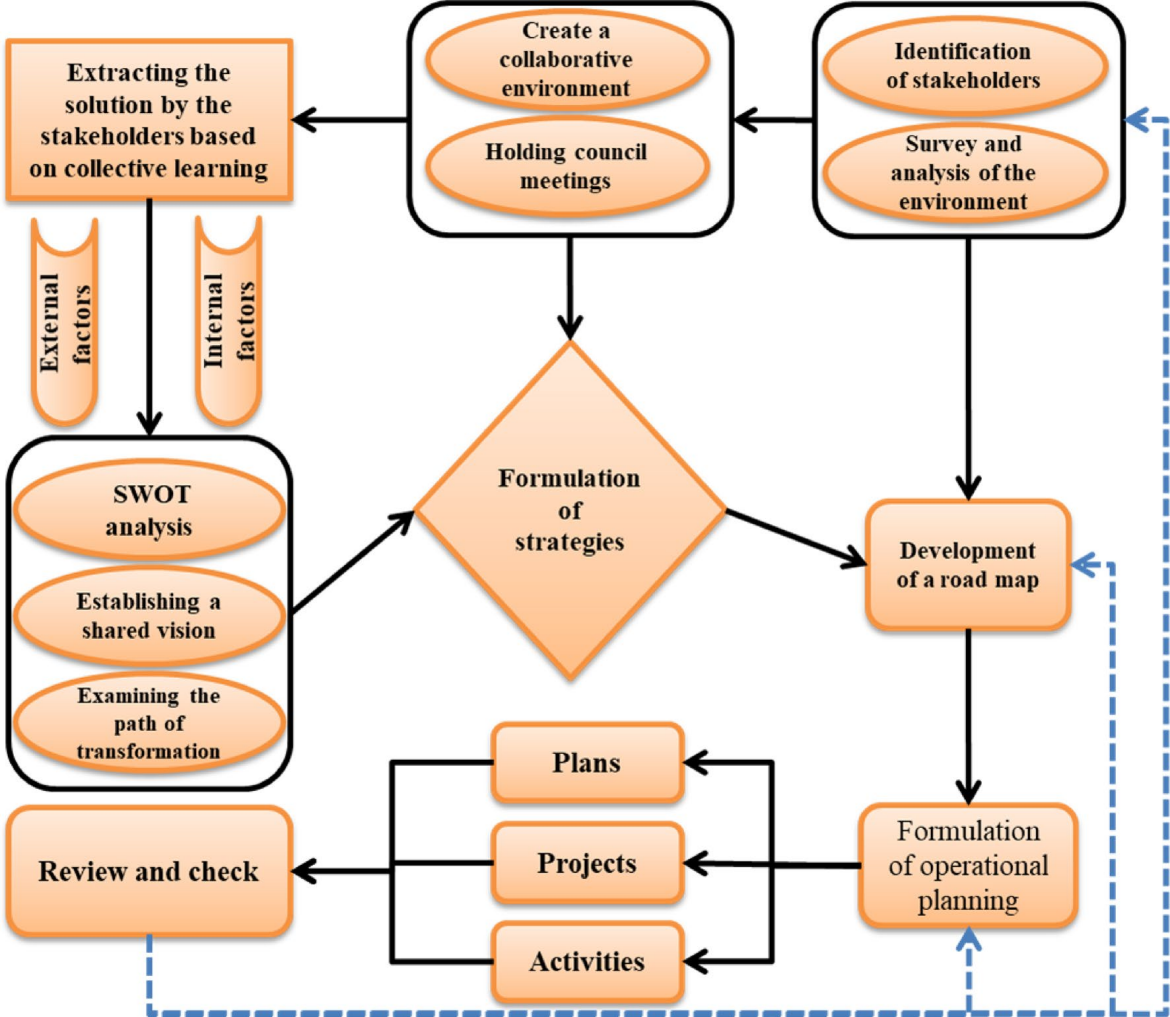
Conceptual framework of the research (Microsoft PowerPoint 2007, https://www.microsoft.com/en-us/microsoft-365/powerpoint).

Figure 2 illustrates the research methodology. Initially, key stakeholders were identified, followed by an examination of the region's issues and problems with their participation. Through participatory sessions, preliminary solutions related to the identified issues and problems were derived. Subsequently, issues and problems were categorized into internal and external factors, and using participatory visioning and strategic management methods, strategies were formulated. The initial roadmap was developed and revised during participatory sessions. Considering that strategic plans are dynamic and require annual review, this plan has been placed on the agenda of the regional water council for continuous evaluation.

A correct understanding of the actors' perspectives regarding the current state of water resource management can enhance their behavior and responsiveness to water resource conflicts, ultimately contributing to integrated water management. The research conducted with the participation of water stakeholders in Mashhad Plain can help identify fundamental water management challenges in this region. Below are some key challenges that may arise in water management in Mashhad Plain:

1. **. Water Scarcity**: One of the fundamental challenges in water management in many regions, including Mashhad Plain, is water scarcity. Population growth, industrial and agricultural development, changes in water consumption patterns, and climate changes can reduce water resources and increase demand.
2. **Climate Changes**: Climate changes can significantly affect precipitation patterns and access to water in Mashhad Plain. Patterns of precipitation, temperature changes, evaporation, reduced rainy days, and increased droughts are some of the impacts that can lead to changes in regional water management.
3. **Land Use**: Changes in land use can also pose challenges in water management in Mashhad Plain. Increasing urbanization, industrial and agricultural development, changes in agriculture patterns, and improper use of water resources can lead to changes in water demand and supply, increasing management issues.
4. **Water Pollution**: Water pollution is also a major challenge in water management. Discharge of industrial water, wastewater, and improper use of fertilizers and chemicals can lead to pollution of rivers and groundwater, threatening the health of groundwater and surface waters.
5. **Coordination and Collaboration Among Stakeholders:** One of the fundamental challenges in water management in Mashhad Plain is coordination and collaboration among various stakeholders. Water stakeholders include government officials, environmental organizations, farmers, industries, local communities, and other related individuals and organizations. For effective water management in Mashhad Plain, it is necessary for all stakeholders to collaborate, share information, and make coordinated decisions.

After identifying the primary issues in water management within the Mashhad Plain, we employed the Snowball Technique to identify key actors who would participate in water management sessions. This technique entails selecting an initial actor and then requesting them to identify and select subsequent actors, and so forth^43^.

These sessions encompassed a total of 4800 person-hours of engagement, with an average of 30 water managers and users participating. They represented a diverse range of organizations, including the Khorasan Razavi Regional Water Company, Mashhad Water and Wastewater Company, Khorasan Razavi Provincial Water and Wastewater Company, Khorasan Razavi Agricultural Jahad Organization, Khorasan Razavi Governor's Office, Mining, Trade, and Industry Organization of Khorasan Razavi, and the General Office of Environmental Protection of Khorasan Razavi, among others. These sessions collectively operated under the banner of the Mashhad Wise Water Forum. The outcomes of these meetings included the identification of goals, available resources, differing viewpoints, and the establishment of a network of connections among the actors. Moreover, these sessions facilitated the extraction of the most suitable policies to address the challenges encountered within the Mashhad Plain.

This article presents the culmination of a several-year endeavor, comprised of several smaller projects, now synthesized by the central research team. Prior related research contributions by Omranian Khorasani^44^, Davary & Omranian Khorasani^45^, Hatami^46^, Salarian^47^, and Davary^48^ have indeed made valuable contributions to various aspects of the research topic. However, none of these previous works possessed the comprehensive dataset and analytical framework presented herein. This article, therefore, offers a unique and comprehensive perspective, consolidating previously fragmented research efforts into a coherent and comprehensive whole, providing a holistic view of the subject matter.

### Analysis of actors

In developing a participatory roadmap for water management in Mashhad Plain, various stakeholders, including local authorities, water consumers' representatives, environmental and agricultural stakeholders, have been identified. Their participation in these sessions ensures representation of diverse perspectives and interests. Through discussions and negotiations, this inclusive approach addresses the concerns and needs of all stakeholders, culminating in a comprehensive roadmap. By engaging individuals and groups with diverse experiences, this participatory method fosters agreement and collaboration, ensuring that the final roadmap reflects collective input and identifies optimal water management strategies for Mashhad Plain.

**Figure 3:**
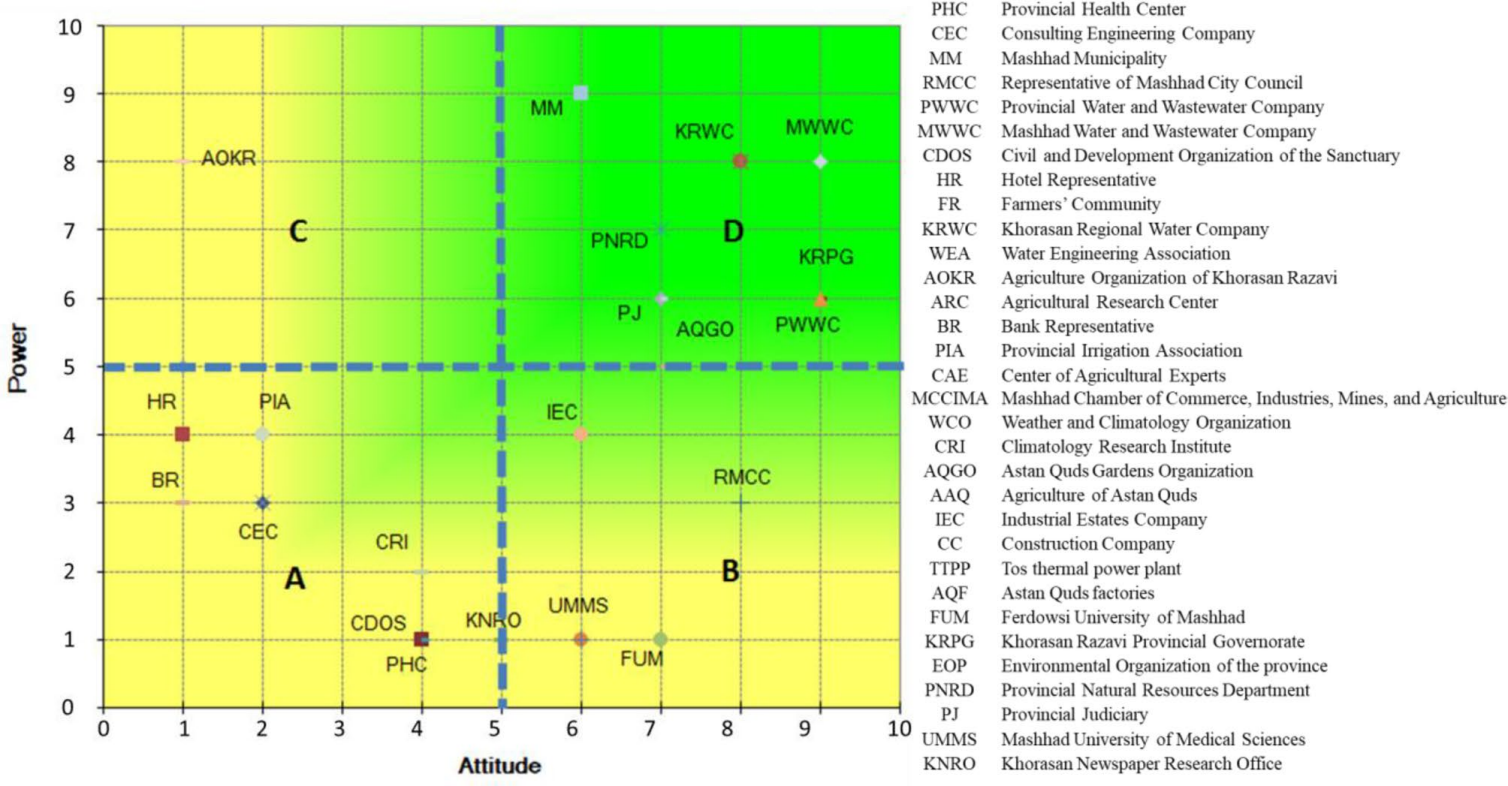
Key actors in Mashhad Plain (Microsoft Excel 2007, https://www.microsoft.com/en-us/microsoft-365/excel).

To assess the effectiveness of actors within the context of Mashhad Plain, we utilized a set of questions that pertained to their managerial decision-making capabilities, power and attitudes. This assessment was carried out with the assistance of a Diagram, as illustrated in Fig. 3. In this chart, Groups in green are key stakeholders.

### Finding a suitable method for interactions

**Figure 4:**
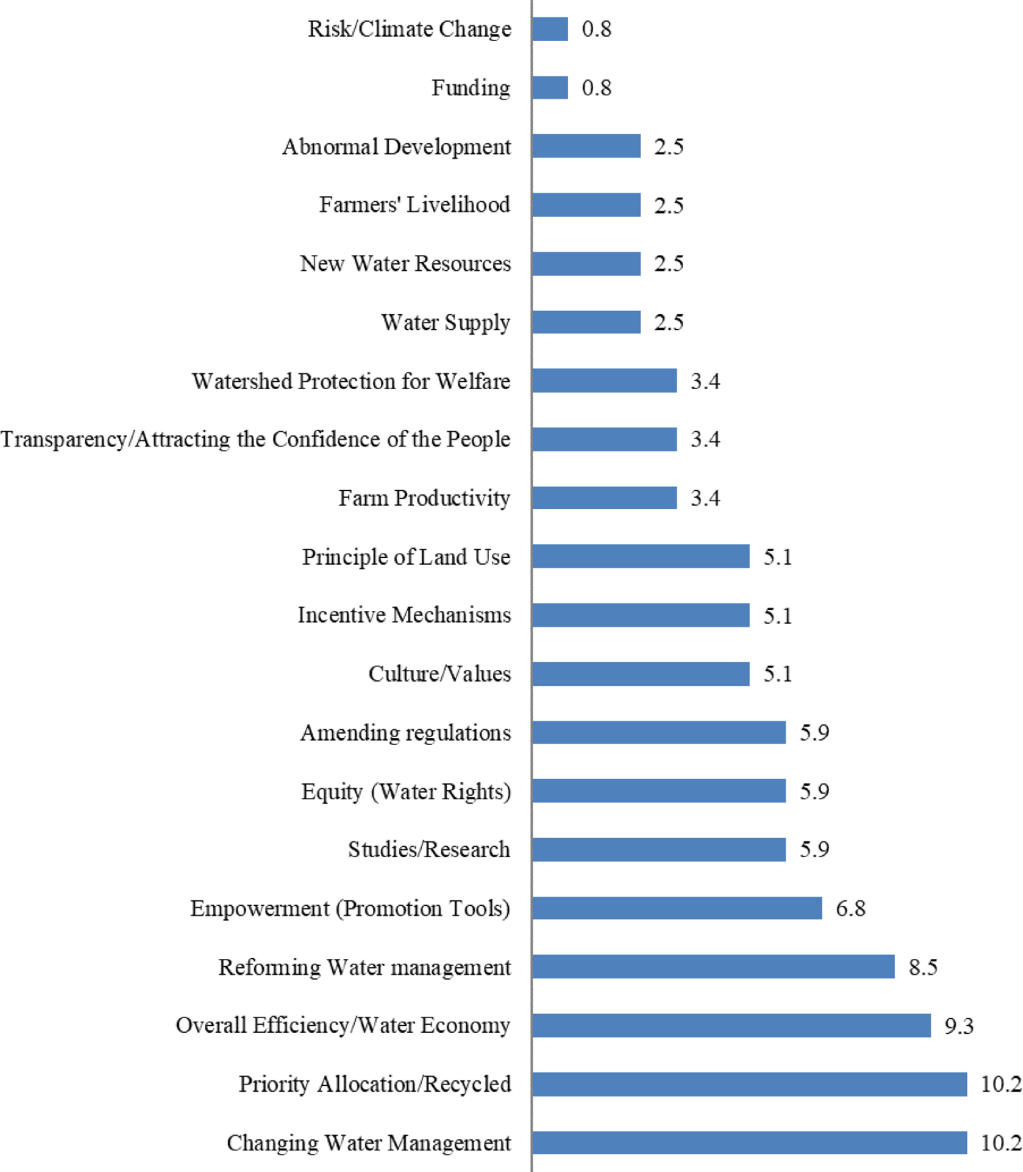
Classification of basic subjects in percent (Microsoft Excel 2007, https://www.microsoft.com/en-us/microsoft-365/excel).

Brainstorming is an individual or group creativity technique in which session participants aim to generate as many ideas as possible on a given issue^49^. During these brainstorming sessions, a total of 118 potential solutions were presented to address the existing water management challenges in Mashhad Plain. Among these, 23 topics were identified as either recurring or closely related concepts and were subsequently consolidated. The remaining 95 initial solutions, proposed by both managers and consumers to enhance the condition of water resources in Mashhad Plain, were further categorized into 20 topics (Fig. 4). The Principle of Land Use entails the coordinated and optimized utilization of land resources while regulating various activities such as agriculture, housing, industry, mining, and environmental conservation. It aims to prevent conflicts and ensure the sustainable use of land, water resources, and natural ecosystems within a specific region. Adhering to this principle is fundamental in natural resource management, urban planning, environmental conservation, and sustainable development. However, these topics encompassed 32 related to comprehensive plain management, 16 associated with allocation management, 28 linked to demand management, and 19 tied to supply management.

The subsequent step involved utilizing the Roadmap tool to develop an integrated conceptual model involving all stakeholders. This model delineates the macro policies for Mashhad’s vision in the year 2041, encompassing six pivotal components: Knowledge, Power, Decision-Making, Order, Attitude, and Cognition. From these policy tiers, a series of solutions were formulated and presented as a macro-management policy sequence. These solutions emerged from 4800 h of participatory meetings conducted within the Mashhad Water Wise Forum. The mechanisms by which the components of the roadmap are addressed are categorized into six distinct groups: skill synergy, reforming rules and regulations, enhancing data systems, reforming economic policies, promoting water culture, and modifying management structure.

### Analyzing the network of relations among activists

Network analysis is an approach that examines organizational and institutional structures, focusing on the impact of social structures on actor relationships^50^. This analysis is grounded in the applied theory of graphs, encompassing the theory of graphs and matrix algebras. Matrices and UCINET software were utilized for data entry and recording, while Graphs and NetDraw software were employed to visualize data and information related to communication patterns. Degree Centrality, Midway Centrality, Proximity Centrality, Eigenvector Centrality, Grouping, and Power Metrics were also applied for both visual and numerical analysis of matrices, aligned with the research hypotheses and objectives (e.g.,^51^). In the network approach, the fundamental principle is to scrutinize the characteristics of relations between and among units, rather than the characteristics of the units themselves. This method transcends the mere description of formal institutional structures, enabling the consideration of key actors in policy-making processes across various domains^52^.

**Figure 5:**
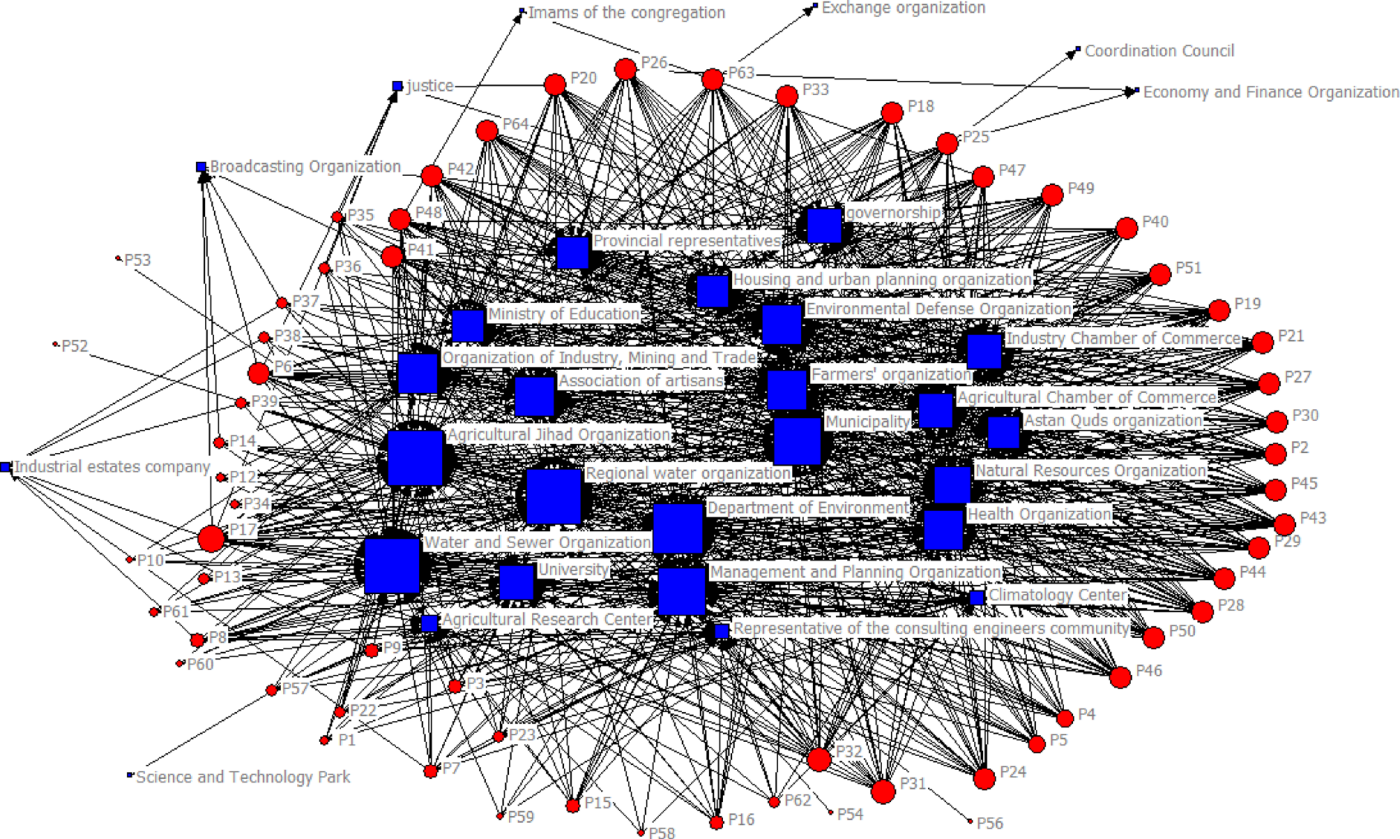
Analysis of the actors’ network (UCINET software, www.analytictech.com).

In this research, the investigation of power centers in water management in Mashhad Plain entailed the observation of formal and informal relations among actors through their interactions and the implementation of macro policies. The process involved several steps:

- Actors were tasked with designating a secretary and executive members for each of the 64 major policies outlined in the Mashhad roadmap.
- To gain insights into the relationships between organizations involved in water management, a paired comparison matrix was generated using UCINET software. The matrix comprised rows and columns representing macro policies and water management organizations in Mashhad Plain, respectively.
- In the third step, numerical values of 0, 1, and 2 were assigned to denote “no communication,” “one-way communication,” and “two-way communication” between the secretaries and executive members of each macro policy in the roadmap.
- Finally, the network of relations and intermediate-level organizations advancing the water management roadmap for Mashhad Plain was visualized using NetDraw software (see Fig. 5).

The concept of intermediate centrality refers to a pivotal actor who holds the utmost influence among all actors involved, as visualized in Fig. 5. It’s important to note that actors are denoted in blue, while the policies integrated into the roadmap are indicated numerically and highlighted in red. This central actor has the capability to regulate the transmission of information, resources, and other essential elements between actors. Essentially, their position signifies that any communication channel between two actors must pass through this key individual. Consequently, this influential actor holds the power to control communication flows among others, disrupt channels, isolate actors, and ultimately exert significant influence. As depicted in Fig. 5, the Regional Water Company of Khorasan-Razavi Province holds a highly centralized position, thus assuming a dominant role in water resource management. This authority possesses the potential to modify the levels of influence and impact exerted by other actors. Serving as a crucial interface among diverse organizations, it has the capacity to facilitate the establishment of an integrated participatory management system, thus emerging as the most influential organization in this context.

### Common cognitive map and conflict resolution

To address conflicts effectively, it is essential to pinpoint areas of convergence and divergence among actors. Conceptual maps and cognitive maps are valuable tools for visualizing mental constructs. In contrast to conceptual maps, cognitive maps can encompass various dimensions of a complex social and economic system, including its environment. Consequently, cognitive maps are highly effective in identifying key issues and exploring future options, particularly when constructed by a group of experts. A roadmap serves as a tool for shaping future water management, and the future itself is a mental construct that can be envisioned in diverse ways by prospective individuals. Therefore, to successfully employ cognitive techniques for forecasting, actors’ cognitive roles are presented within the context of a comprehensive roadmap for water resource management in Mashhad Plain. The richness and dynamism of an individual’s cognitive map often correlate with their level of experience with a given environment or event.

A human's cognitive map reflects the contents of their mind. Essentially, wisdom can be regarded as an extraction from the contents of an individual’s cognitive map, obtained through self-conscious and unconscious processes. What is crucial here is the identification and mapping of roadmap components, as well as determining the desired content for the envisioned future. Individual cognitive analyses enabled the identification of distinctions and commonalities among actors. Each person’s cognitive map was derived primarily from the roadmap, pinpointing areas of consensus (hot spots) and points of divergence (challenges) for each actor. This analysis was conducted at the cluster and group levels for both managers and consumers. After analyzing individual cognitive maps and aggregating them, the points of convergence and divergence were synthesized. From this point onward, greater emphasis was placed on understanding the differing viewpoints among actors.

The roadmap for water management in Mashhad, representing a consensus among a group of managers and consumers, served as the foundational model for “conflict resolution” in addressing the water crisis. To achieve greater cohesion and alignment among actors, this roadmap was subsequently refined following a meticulous review by actors, who identified and prioritized macro policies with precision and comprehensiveness. Subsequently, six elements of the roadmap underwent evaluation through meetings and questionnaires distributed to 40 key actors (see Table 1). Within this table, “usefulness” and “probability” denote the effectiveness of policies and the likelihood of successful implementation, respectively.

**Table 1:** A portion of the questionnaire for roadmap analysis based on the severity and likelihood of policy implementation.

Vision: On the horizon of 2041, Mashhad is a Plain with a stable equilibrium in water resources and consumptions			
The table assessing Mashhad’s water prospecting strategies and the following macro policies			
1. Integrated management of water resources and consumption has been established in the Mashhad study area; (Approved)		Evaluation criteria (1 to 9)	
Row/Priority	Description of Macro Policy	Effectiveness	Likelihood
Macro policy 1	Integrated allocation of water resources based on renewable and transferable water to ensure a sustainable water resource balance (and review of total allocated allocations) (Systematic Thinking)		
Macro policy 2	Formation of water councils with the participation of farmers at the watershed/city/province levels		
Macro policy 3	Increasing water resources through external supply, balancing operations, watershed management, aquaculture, using modern methods, virtual water, etc		

2. Participation of stakeholders (beneficiaries and legal entities) in the management of water resources at all levels is organized and regulated (approved)		Evaluation criteria (1 to 9)	
Row/Priority	Description of Macro Policy	Effectiveness	Likelihood
Macro policy 1	Identification and gradual organization of stakeholders: from water user associations at each uptake point to the Syndicates at the catchment area		
Macro policy 2	Identification and gradual, proportionate granting of responsibility and authority to water user associations		
Macro policy 3	Training, Empowerment, and Cultural enhancement of the water users (Water Companies and Authorities) (Proportional to Macro Policy)		

The primary outcome of the roadmap sessions was consensus among experts. When experts from different groups attend roadmap sessions and engage in negotiation and discussion, it signifies that the roadmap has been designed based on diverse experiences and perspectives. This process may take hours and indicates the attention and effort put into reaching a final agreement. However, it's essential to note that achieving consensus among experts and final agreement on the roadmap are only initial steps. For a successful implementation of the roadmap, other factors such as resource allocation, coordination among members, project management, and continuous performance evaluation are crucial. In other words, consensus among experts alone does not guarantee complete implementation; instead, a successful roadmap execution requires attention to implementation details and proper management. Therefore, while considering expert consensus as a significant phase in roadmap design and execution, utilizing suitable resources and processes for its full and successful implementation is paramount.

**Figure 6:**
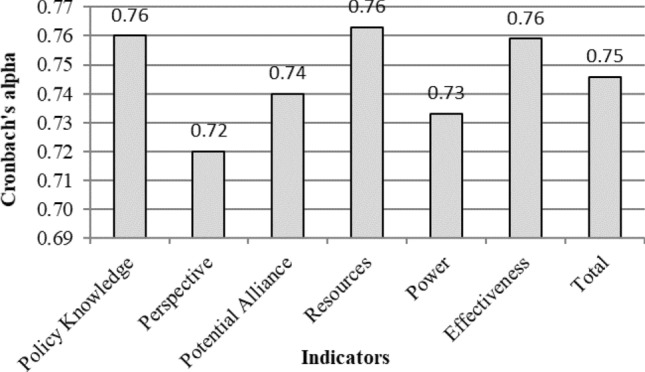
Reliability of the questionnaire (Microsoft Excel 2007, https://www.microsoft.com/en-us/microsoft-365/excel).

The reliability of the questionnaire was assessed using Cronbach’s alpha test, a quantitative analysis method for evaluating indicators^53^. Cronbach’s alpha values above 0.7 indicate good correlation and agreement among experts, values between 0.5 and 0.7 signify moderate correlation, while values below 0.5 suggest poor correlation and agreement, necessitating potential reevaluation. The Cronbach's alpha values, as shown in Fig. 6, indicate that the questionnaire exhibited acceptable reliability.

Using cognitive mapping, it is possible to aid in reducing water consumption by consumers in the Mashhad Plain. The strategies employed to reduce water consumption in the Mashhad Plain are detailed below:

1. Understanding Water Management Challenges: Through cognitive mapping, efforts have been made to identify the challenges faced by water managers and consumers in the Mashhad Plain. This understanding enables the identification of areas and activities with higher water consumption in the region. By recognizing these patterns, programs and policies can be designed to help reduce water consumption in these areas.
2. Reflecting on Perspectives and Beliefs: Analyzing cognitive maps allows for an understanding of the perspectives and beliefs of water managers and consumers in the Mashhad Plain regarding water. This insight is crucial for designing effective campaigns and water education initiatives. Understanding consumers' perspectives and beliefs can facilitate the design of educational content and information to encourage water conservation based on these viewpoints.
3. Enhancing Awareness: Using cognitive mapping, weaknesses and misconceptions in the knowledge of water managers and consumers in the Mashhad Plain about water can be identified. By providing accurate information and appropriate training, awareness and knowledge among consumers about proper water management can be increased.
4. Designing Effective Environments: Through cognitive mapping analysis, water managers and consumers in the Mashhad Plain can gain a better understanding of environmental factors influencing water consumption. These factors may include water supply, infrastructure, laws, regulations, etc. By designing and improving these environmental factors, it is possible to outline environments for the water management roadmap in Mashhad.

Cognitive maps of actors were derived from the responses, illustrating how actors interacted in terms of Attitude, Power, Cognition, Knowledge, Order, Power, and Decision. The outputs of this cognitive phase, which encompassed shared and differing points among actors, were analyzed as follows:

(A) Challenging points and even neutral points within the roadmap were extracted for each actor, based on the severity and likelihood.
(B) Analysis of shared and differing points at the organizational group level of actors was conducted, leading to the extraction of solutions for consensus-building or conflict reduction.
(C) Building upon the proposed solutions, the fundamental water management model was refined, culminating in the final roadmap for water management in Mashhad.

Cognitive map charts were generated using Origin Pro 2016 software. The model inputs consisted of macro policies developed within the framework of the Mashhad Wise Water Forum, while the outputs comprised the cognitive maps of actors encompassing the six components.

Finally, this study employed a participatory approach to devise the roadmap for water management. Engagement of experts from diverse groups in dedicated sessions facilitated the accumulation of varied viewpoints and perspectives. Drawing from these diverse experiences and perspectives, comprehensive management strategies were formulated. This research marks a significant stride in strategic management, notably for its consideration of a wide array of experiences and viewpoints in strategy design. Perspectives and concerns of pivotal stakeholders, individuals lacking prior strategic management experience, were collected and expertly transformed into actionable strategies. Throughout stakeholder participation in sessions, strategies underwent refinement and enhancement using participatory methodologies. A notable accomplishment of this research lies in the establishment of participatory sessions engaging representatives from all sectors, culminating in the development of a collaborative roadmap. Particularly in areas where consumer involvement in water management is unfeasible, the creation of such participatory sessions becomes pivotal. These sessions fostered discussions and negotiations among diverse stakeholders, contributing to the formulation of a unified roadmap for water management in the region. Additionally, the application of mind mapping as a tool to comprehend and evaluate diverse perspectives on the roadmap proved highly effective. This method allowed us to gain insight into genuine viewpoints while mitigating attempts by policymakers to deceive the public or consumers due to concerns of interest loss. The amalgamation of participatory methodologies and mind mapping in this research has significantly contributed to establishing a unified perspective on water management in the Mashhad region.

## Results

**Figure 7:**
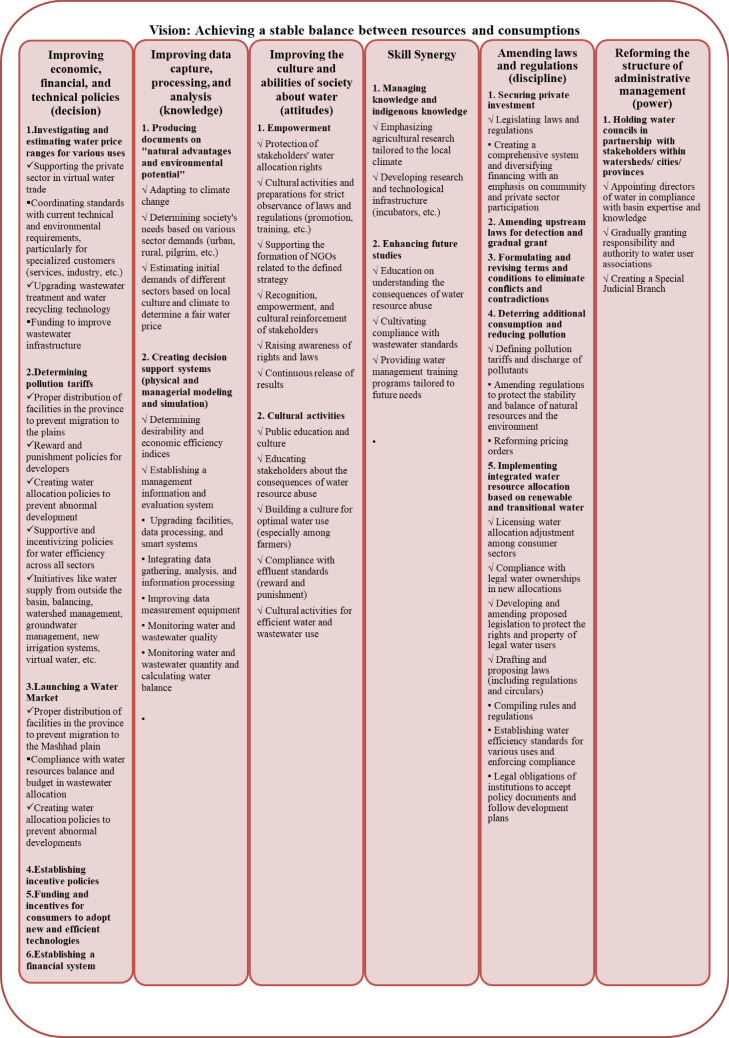
Road map of water management in mashhad plain and presentation of its six key components (Microsoft PowerPoint 2007, https://www.microsoft.com/en-us/microsoft-365/powerpoint).

Figure 7 illustrates the path to achieving sustainable equilibrium in resource allocation and water consumption in the Mashhad Plain. The roadmap systematically breaks down the ultimate goal into layers or levels, allowing managers and consumers to envision policies, costs, and horizons for each water management component. A comprehensive grasp of the six-axis components of the roadmap serves as a fundamental requirement for managing water resources in the Mashhad Plain. The roadmap generated for each component represents a logical sequence of macro policies aimed at achieving the ultimate goal. The sequencing of macro policies on the roadmap addresses two fundamental questions: how policies are implemented and why policies should be implemented. Additionally, the logical order of policies, in terms of realization, is presented from left to right in each graph. Contradictions became apparent after designing a common conceptual model agreed upon by all actors, as encapsulated in the water management roadmap of the Mashhad Plain. This shared conceptual model helps dispel potential misunderstandings about water management issues.

Due to constraints in paper publication, it is not possible to mention all macro policies or even the executive policies within the roadmap. Under these circumstances, some of the macro policies specified in your research roadmap are clearly and thoroughly observable and examinable. It should be noted that the roadmap, which is the primary output of this research, covers specific macro policies and has listed them explicitly. This roadmap can serve as a reliable source for understanding the relevant policies. Therefore, in these circumstances, utilizing the roadmap as the primary output of the research and harnessing the information outlined within it can be a suitable solution for water management, ensuring the sequence and coherence of the programs.

**Figure 8:**
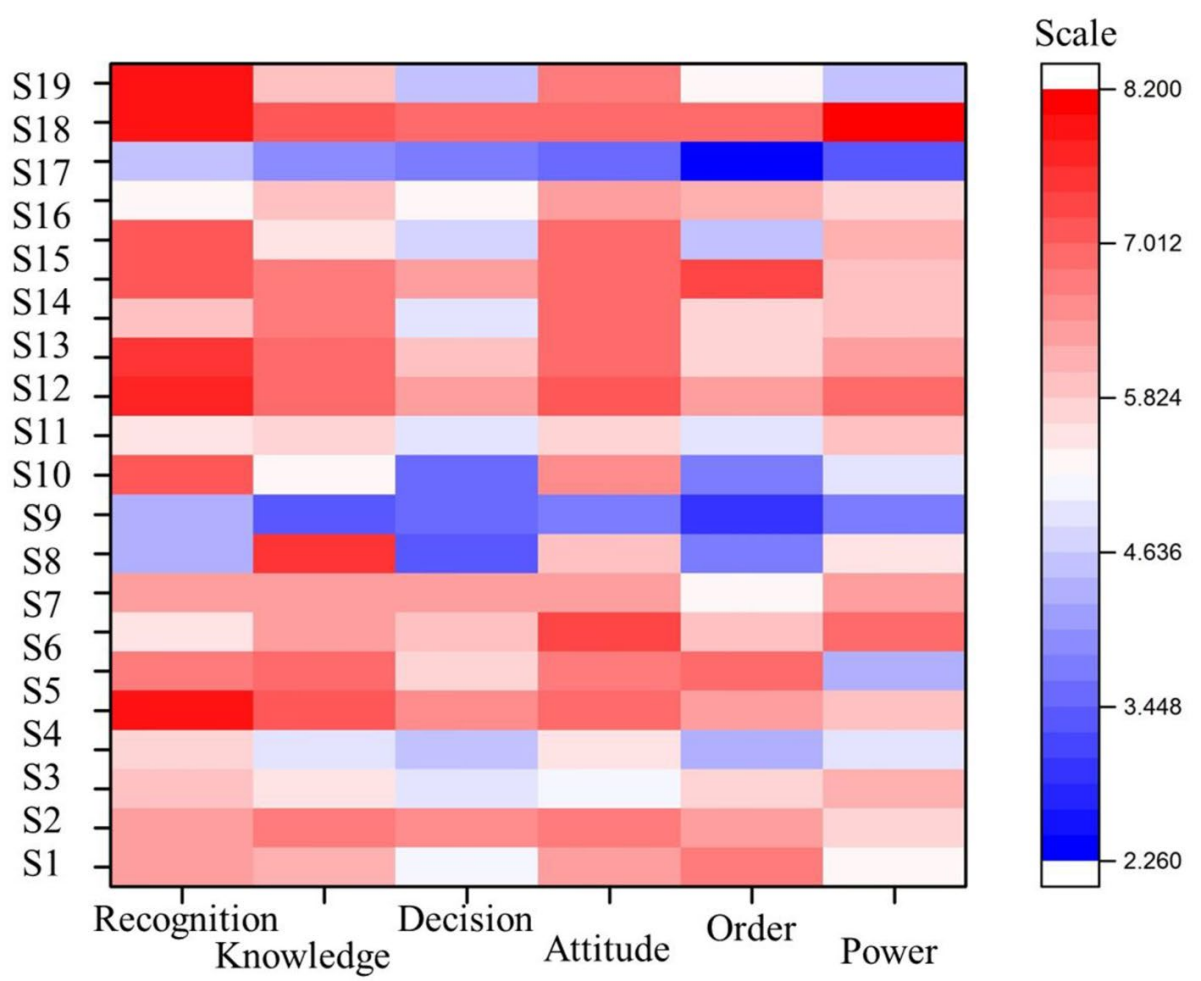
Cognitive map of actors based on different component (Red points on the map indicate the highest convergence, while blue points represent lower convergence and differing opinions) (Origin software 2018, https://www.originlab.com).

Following the collection of questionnaires based on Table 1 and the assessment of questionnaire reliability, cognitive maps of activists were derived (Fig. 8). The differences in actors' responses, denoted as points of convergence and divergence, are illustrated in cognitive map diagrams based on the likelihood of policy occurrence and the perceived usefulness of major water management policies in the Mashhad Plain. Each part corresponds to one of the six components: “Attitude,” “Cognition,” “Knowledge,” “Order,” “Power,” and “Decision” outlined in the roadmap. The X-axis and Y-axis represent macro policies (questions) and actors from Mashhad's roadmap, respectively. The third axis (scale) quantifies the product of the probability of policy occurrence and its perceived usefulness from the actors' perspectives, represented by colored dots. Greater diversity in colors signifies a wider range of viewpoints.

**Figure 9:**
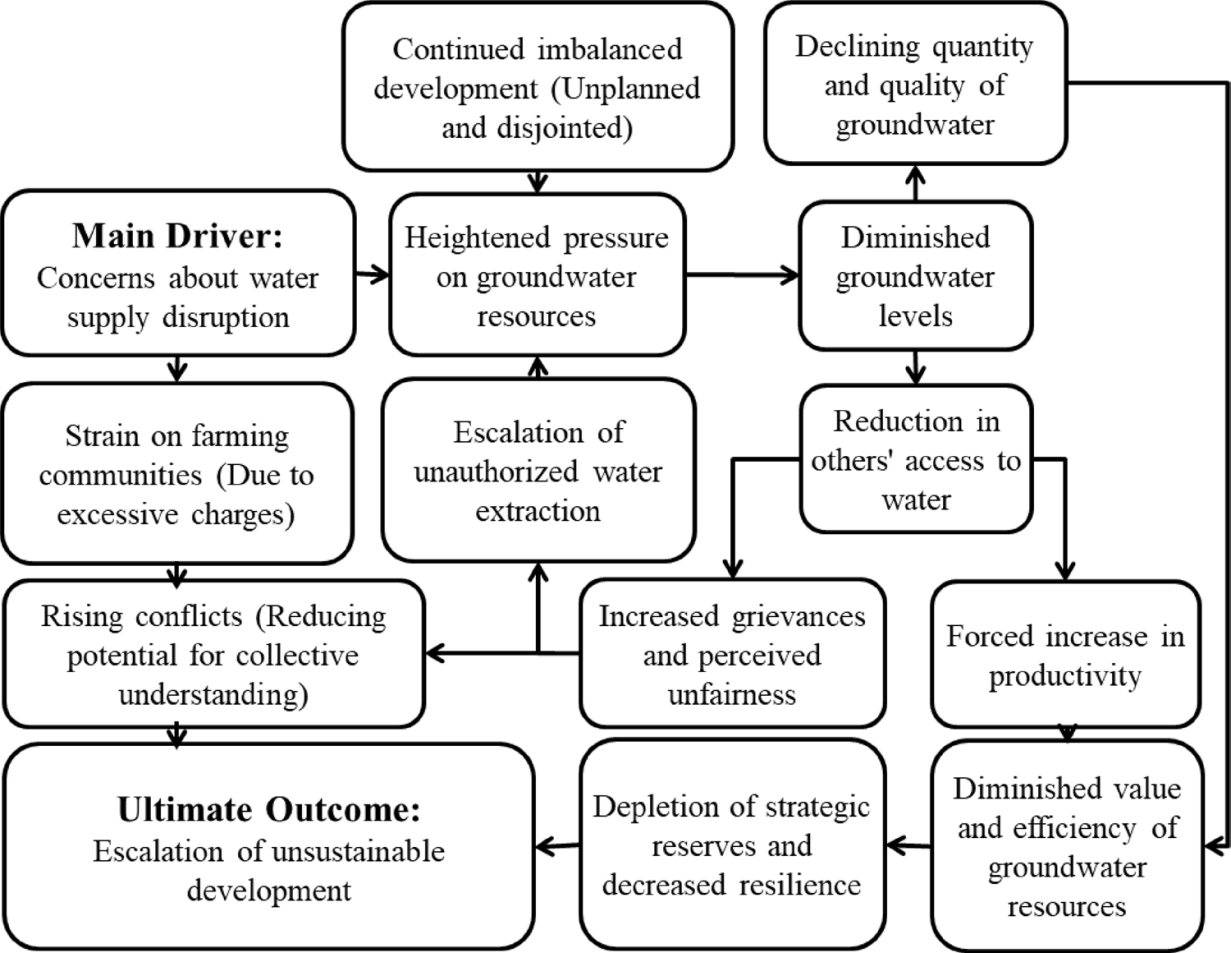
Causal relationships of water managers in Mashhad Plain cognitive map (Microsoft PowerPoint 2007, https://www.microsoft.com/en-us/microsoft-365/powerpoint).

The paths to improving water management are depicted based on the six-axis components of the roadmap. As indicated by Fig. 8, cognitive maps of actors exhibit minor challenges in all cases. However, in the components of “Decision,” “Order,” and “Power,” the diversity of colors indicates differences in actors’ opinions compared to other components. Blue points represent macro policies deemed to have lower “probability of occurrence” and “usefulness” from the actors’ perspective. The cognitive map of consumers (members X18 and X8) reveals that the largest water users, particularly in the agricultural sector, hold a more pessimistic view of the water management roadmap in the Mashhad Plain. The causal relationships within the cognitive map of managers are complex, given their comprehensive responsibility areas. Consequently, their opinions encompass numerous factors and their interrelations. As depicted in Fig. 9, managers’ primary concern centers on enhancing the quality and quantity of water resources in the Mashhad Plain. Furthermore, Fig. 9 illustrates the causal relationship between the cognitive maps of water consumers in the Mashhad Plain. Understanding the correlation between farmers’ concerns about their livelihoods and unauthorized wells is essential to reduce their vulnerabilities. Farmers aim to safeguard their livelihoods by identifying unauthorized wells in the plain.

The analysis of causal relationships among actors revealed that water managers have, for unknown reasons or due to the perceived high cost, seemingly disregarded the livelihoods of farmers. They have opted for a seemingly simpler solution—reducing farmers’ water rights—to alleviate pressure on water resources in the Mashhad Plain. Unfortunately, such simplified approaches only address the symptoms of the problem, leaving the root issue unresolved. Over time, this core problem worsens, and the system loses its capacity to address the water deficit in the province. Understanding these processes can enhance both managers' and consumers' comprehension of system dynamics. Ultimately, this process can lead to a conscious consensus between consumers and water managers and increase public awareness regarding water management decisions in the Mashhad Plain.

## Discussion

Insufficient planning and disregard for cultural, social, economic, and legal factors can result in severe water management issues, evident in Mashhad Plain and other Iranian regions. Effective water management demands comprehensive planning encompassing these multifaceted aspects, emphasizing collaboration among diverse stakeholders. The problems in Mashhad Plain arise from inadequate planning, reckless water resource utilization, and environmental negligence, exacerbated by organizational and stakeholder coordination lapses. Therefore, a more thorough and inclusive approach to water resource management is imperative. This approach should involve assessing potential impacts, optimizing water resource utilization, promoting prudent water consumption practices, and environmental preservation, addressing societal needs. In line with this, our research aims to create a detailed water management roadmap, considering all facets of water management.

The findings of this study shed light on the complexities and nuances of water resource management in the Mashhad Plain, a region grappling with pressing challenges related to water scarcity, conflicting interests, and sustainable resource use. By employing a comprehensive methodology that encompasses actor analysis, cognitive mapping, and the development of a roadmap for water management, this research has advanced our understanding of the intricacies involved in addressing these multifaceted issues^54^.

**Key actors and power dynamics:** The identification of key actors is a pivotal outcome of this study. The prominence of the Khorasan-Razavi Regional Water Company and the Mashhad Water and Wastewater Company as influential actors underscores the pivotal role of these organizations in shaping water policies and resource allocation within the region. Their influence extends beyond mere participation, with the Khorasan-Razavi Regional Water Company, in particular, emerging as the linchpin that can potentially facilitate the development and implementation of integrated participatory water management. However, it is equally vital to acknowledge the Company for Industrial Towns as the most impacted actor, as it reflects the broader implications of water management decisions on various sectors, including industry. Additionally, the Agricultural Experts Panel, representing the farming community, assumes a dual role as highly influential and impacted, signifying the delicate balance between agricultural livelihoods and sustainable water resource management.

**Conflict resolution and cognitive mapping:** The utilization of cognitive mapping has proven to be an invaluable tool for discerning the divergent perspectives of different actors within the region. A striking revelation from the cognitive maps is the incongruity in priorities between consumers and managers. While consumers primarily emphasize the preservation of livelihoods, managers place a greater emphasis on enhancing water resource quality and reducing agricultural water consumption. This misalignment poses a significant challenge in achieving consensus and highlights the urgent need for reconciling these conflicting priorities. The existence of conflicts, both minor and serious, in a substantial portion of the macro policies underscores the complexity of water management decision-making. It is evident that navigating these disputes requires a nuanced approach that takes into account the unique viewpoints and concerns of each actor.

**The roadmap for water management:** The development of a comprehensive roadmap for water management represents a significant milestone in this study. The roadmap systematically dissects the ultimate goal of sustainable water resource management into layers, offering a clear vision of policies, associated costs, and future horizons for each component of water management. By delineating the logical sequence of macro policies, the roadmap guides stakeholders on the “how” and “why” of policy implementation. Crucially, this roadmap serves as a unified conceptual model, fostering a shared understanding of water management among actors. It has the potential to align disparate viewpoints, encourage transparent discussions, and facilitate the identification of win–win solutions. However, the success of this roadmap hinges on the ability to address the fundamental conflicts and disparities uncovered by cognitive mapping.

**Future directions:** As a logical extension of this research, the development of a dynamic system model, integrating both cognitive and roadmap components, offers a promising path forward. Such a model can simulate the consequences of different policy decisions, providing valuable insights into their long-term impacts on water resource management and potential conflict resolution.

Overall, this study has provided a comprehensive framework for understanding and addressing the water management challenges in the Mashhad Plain. By illuminating the key actors, power dynamics, cognitive disparities, and the roadmap for water management, it offers a roadmap of its own—a roadmap to achieving a more sustainable and harmonious future for water resource management in the region. It is imperative that these findings inform future policy decisions, fostering cooperation and consensus among all stakeholders to effectively address the pressing water issues faced by the Mashhad Plain.

## Conclusion

The exploration of key actors in the Mashhad Plain has unveiled pivotal contributors to water resource management. Notably, the Khorasan-Razavi Regional Water Company and the Mashhad Water and Wastewater Company emerge as influential players, while the Company of Industrial Towns experiences significant impacts. In representation of the farming community, the Agricultural Experts Panel holds sway both as an influential force and as a group deeply affected by water management decisions. An intricate network analysis within the Mashhad Plain delineates the Khorasan-Razavi Regional Water Company as a central figure with substantial authority over water resource management, indicated by its highest intermediate centrality. This prominence designates it as a cornerstone in the orchestration of integrated participatory water resource management. Subsequently, a collaborative endeavor among diverse stakeholders in numerous meetings yielded 118 potential solutions, with 64 identified as macro policies. These macro policies culminated into a comprehensive roadmap, structured into six components: “Attitude,” “Cognition,” “Knowledge,” “Order,” “Power,” and “Decision.”

Throughout this study, the cognitive maps of involved stakeholders have illuminated divergent perspectives concerning water issues and the groundwater crisis. Consumers underscore the significance of “livelihoods,” while managers prioritize the enhancement of water resource status and the reduction of water consumption within the agricultural sector. Consequently, the reduction in agricultural water usage seems to counteract the interests of the farming community. Among all stakeholders, farmers exhibit the most pessimistic views regarding the roadmap for Mashhad Plain Water Management.

The construction of cognitive maps for stakeholders proves invaluable in shedding light on conflicts within water resource management in the Mashhad Plain. These cognitive maps offer insights and solutions for resolving intricate issues related to water management. As indicated by the Mashhad cognitive map, stakeholders exhibit significant conflicts in four cases (6%), minor conflicts in 18 cases (30%), and no conflicts in the remaining macro policies. To provide a more profound understanding of the repercussions stemming from the implementation of the proposed decisions and policies, we recommend developing a dynamic system model that employs the cognitive maps and roadmap presented in this study. This approach facilitates a more comprehensive assessment of the implications of decisions in resolving conflicts related to water management.

The development and analysis of cognitive maps play a substantial role in conflict resolution within the sphere of water resources. Cognitive maps serve as visual tools for depicting the perspectives, opinions, and objectives of distinct individuals and groups in a structured and graphical format concerning a specific issue. Grounded in past experiences, priorities, and values, cognitive maps enhance our comprehension of individuals' viewpoints and requirements, consequently enabling a more effective approach to conflicts in water resource management. In essence, the use of cognitive maps for conflict resolution in water resource management lies in their capacity to offer access to diverse perspectives and opinions, fostering a shared platform for discourse and exchange. Through cognitive map analysis, relationships among individuals and groups become transparent, and areas of potential conflict and consensus emerge. This understanding aids in devising strategies for conflict resolution and establishing common ground among varied stakeholders.

A deeper comprehension of diverse perspectives and goals fosters the formulation of apt strategies and policies for water resource management. In the case of the Mashhad Plain, cognitive mapping facilitates the identification of the perspectives and needs of assorted groups involved in decision-making and water resource management. These groups encompass farmers, industries, government officials, local communities, and other relevant organizations and individuals. By generating cognitive maps, the multitude of viewpoints and preferences regarding water resource utilization become visible, ultimately deepening the understanding of the water-related challenges and conflicts within the Mashhad Plain.

Network analysis and integrated water resource management, while distinct concepts, converge to enhance water resource management. Network analysis entails the exploration of complex network structures and dynamics, whereas integrated water resource management is concerned with the efficient oversight of diverse water resources within a region. Network analysis assists in comprehending the dynamics and interplay among different stakeholders.

In the realm of water resources, stakeholders encompass individuals, organizations, industrial and agricultural entities, environmental bodies, and more. Employing network analysis, the relationships among these stakeholders are unveiled, shedding light on their impacts and interactions within the sphere of water resources. Network analysis demonstrates how alterations in relationships among different organizations and groups can enhance the distribution and utilization of existing water resources or how conflicts among various stakeholders can lead to fitting solutions for optimal water resource management.

The application of network analysis holds the potential to enhance integrated water resource management. A more profound understanding of the network's dynamics and relationships empowers the formulation of strategies to boost coordination among stakeholders, thereby facilitating improvements in regional and international water resource management. By discerning the network's strengths and weaknesses, solutions can be identified to heighten efficiency and efficacy, ultimately contributing to enhanced integrated water resource management.

Furthermore, to enhance the state of water resources in the Mashhad Plain, specific strategies and interventions are proposed within the Water Resources Management Roadmap. These strategies and interventions hold the promise of elevating both the quality and quantity of water resources, improving efficiency, fostering environmental conservation, and enhancing coordination among stakeholders. It’s important to acknowledge that the success of these strategies and interventions hinges on various factors such as financial resources, stakeholder collaboration, and technical support.

Here are several illustrations of strategies and interventions suggested in the Water Resources Management Roadmap for enhancing the condition of water resources in the Mashhad Plain:

1. **Sustainable Agriculture Enhancement:** This strategy entails elevating water efficiency in the agricultural sector, optimizing irrigation systems, and implementing effective water resource management practices in agriculture. The outcomes include reduced water wastage, improved water quality, and increased crop production.
2. **Demand Management:** This strategy revolves around raising public awareness about responsible water management, encouraging optimal water use by individuals and organizations, promoting a culture of water conservation, and establishing effective water resource management policies. These measures lead to reduced water demand, enhanced water distribution, and water resource preservation.
3. **Development of Water Infrastructure:** This strategy encompasses the construction and enhancement of water-related infrastructure, including dams, reservoirs, irrigation and drainage networks, water treatment facilities, and more. By establishing robust and efficient water infrastructure, a reliable water supply, improved water distribution, and enhanced water utilization efficiency can be achieved.
4. **Water Resource Protection and Optimization:** This strategy addresses the protection of surface and groundwater sources, optimization of water usage, and the conservation of watershed areas. Measures include preventing water source pollution, preserving watershed regions, enhancing fisheries management, and safeguarding aquatic biodiversity. This strategy leads to water resource preservation, improved water quality, and environmental conservation.

These strategies and interventions have the potential to yield various positive impacts on water resource management in the Mashhad Plain, such as:

- **Improved Water Distribution:** Enhanced management and coordination among various stakeholders, along with the optimization of water resource utilization, can lead to improved water resource distribution in the Mashhad Plain, benefiting agriculture, industry, and urban areas.
- **Increased Water Efficiency:** Improved water infrastructure, efficient water usage across different sectors, public awareness about water conservation, and reduced water losses lead to increased water efficiency and minimized wastage.
- **Water Resource Conservation:** Measures aimed at safeguarding water sources, preventing water pollution, preserving watershed areas, and maintaining aquatic biodiversity contribute to the conservation of water resources.
- **Mitigation of Drought and Water Scarcity**: Enhanced water management and optimal resource utilization can reduce the frequency and impact of droughts and water shortages, enhancing the region's resilience to climate changes.

In summary, the Water Resources Management Roadmap for the Mashhad Plain aspires to elevate the quality and quantity of water resources, augment efficiency, promote environmental conservation, and enhance coordination among stakeholders. By implementing the outlined strategies and interventions, the state of water resources can be improved, leading to more effective and sustainable water resource management.

## Data Availability

The datasets used and/or analysed during the current study available from the corresponding author on reasonable request.
